# Editorial: Yoga to promote mental health in occupational health settings

**DOI:** 10.3389/fpubh.2024.1460432

**Published:** 2024-07-29

**Authors:** Apar Avinash Saoji, Venugopal Vijayakumar, Kaushik Chattopadhyay, Elisa Harumi Kozasa

**Affiliations:** ^1^School of Yoga and Naturopathic Medicine, Division of Yoga and Life Sciences, Swami Vivekananda Yoga Anusandhana Samsthana, Bangalore, India; ^2^Department of Yoga, Government Yoga and Naturopathy Medical College, Chennai, India; ^3^School of Medicine, University of Nottingham, Nottingham, United Kingdom; ^4^Hospital Israelita Albert Einstein, São Paulo, Brazil

**Keywords:** yoga, holistic approach, mental health promotion, occupational hazard, physical and psychological occupational health

Work-related stress is the reaction people may experience when faced with work demands and pressures, causing them to feel overwhelmed. Stress is often exacerbated when employees perceive a lack of support from their supervisors and coworkers and feel they have little control over their work tasks (1).

Yoga, an ancient science that originated in the Indian subcontinent, has become popular for its health benefits. Yoga aims at self-realization and includes practices such as *yama* and *niyama* (self-discipline), *shatkarma* (six cleansing practices), *asana* (physical postures), *pranayama* (regulated breathing), *dharana* (concentration), and *dhyana* (meditation) (2).

Promoting health and enhancing wellbeing in the workplace can be achieved through practicing yoga. A systematic review has shown that yoga has positive impacts, particularly in stress management. Notably, no adverse effects were reported in the review (3). An evidence map, which provided an overview of research on mindfulness interventions in the workplace, supported the enhancement of employee health, wellness, and performance. Potential positive outcomes were presented for chronic illness, substance use, depression, pain, anxiety, perceived stress, and somatization, among others (4).

The current special topic focuses on the impact of yoga practices in occupational health settings, featuring articles that address a range of issues using various study designs. In this Research Topic, Bhardwaj et al. compared healthcare providers in a mHealth-aided 12-week yoga-based meditation and breathing intervention to a waitlist control group. After 12 weeks, the within-group analysis revealed significant improvements in the Maslach burnout inventory and professional quality of life outcomes for the experimental group. In a yin yoga intervention delivered online, Somere et al. found improvements in anxiety levels after 10 weeks compared to a control group.

Qualitative studies provide deeper insights into participants' opinions and experiences. Hagen and Hagen investigated how yoga could facilitate employees' wellbeing and ability to cope with stress. Qualitative interviews with regular yoga practitioners revealed that they experienced yoga positively and that it reduced their job-related stress.

Cyclic meditation is a form of moving meditation which improves sleep quality (5). Paranthatta et al. compared a group receiving 45 min of cyclic meditation, 7 days a week for 3 weeks, to a control group who continued with their routine activities. This study assessed the impact of cyclic meditation among sailors on board merchant ships. The cyclic meditation group showed improvements in sleep, blood pressure, pulse rate, and anxiety.

In a comprehensive survey, Zok et al. found that yoga forms incorporating fluid movements and synchronized breathing techniques may help manage stress.

The manuscripts submitted to this Research Topic but rejected typically lacked a control group when determining effectiveness or had other methodological issues, such as randomization, lack of valid assessment tools, etc. Therefore, we recommend carefully designing future studies to address these methodological concerns. Through this Research Topic, we aimed to bring new evidence of the application of yoga, including meditation, in occupational health settings. We hope that the readers appreciate the evidence presented on this topic.

## Author contributions

AS: Writing – review & editing, Writing – original draft, Supervision, Resources, Project administration, Methodology, Conceptualization. VV: Writing – review & editing, Writing – original draft, Conceptualization. KC: Writing – review & editing, Writing – original draft. EK: Writing – review & editing, Writing – original draft.
